# The Rare Occurrence of a Left Atrial Thrombus in a Dog

**DOI:** 10.1155/2020/8848627

**Published:** 2020-10-22

**Authors:** Sven Otto, Robert Höpfner

**Affiliations:** ^1^Tierklinik Panitzsch, Carl-Benz-Straße 2, 04451 Borsdorf, Germany; ^2^Kleintierspezialisten Berlin, Wittestraße 30 Haus P, 13509 Berlin, Germany

## Abstract

Intracardiac thrombi are rare in dogs. If they occur, they are mostly seen in the right atrium while only two case reports describing a mural left atrial thrombus in dogs are available. This case report describes a 14-year-old mixed-breed bitch that was presented at the clinic because of dyspnoea. The dog suffered from alopecia for about three years and displayed polyuria and polydipsia for a number of months. Clinical examination revealed intensified inspiratory and expiratory respiratory sounds and various heart sounds. Chest X-ray showed cardiomegaly (VHS 11) and a mixed bronchoalveolar lung pattern of the caudal lung. A transthoracic echocardiography was performed and showed a moderate mitral regurgitation, a highly dilated left atrium, a low-grade pulmonary insufficiency, and a minimal aortic and tricuspid insufficiency. Additionally, a free-floating ball thrombus was found in the left atrium. Hyperadrenocorticism was indicated by an ACTH stimulation test. The previous medical therapy of the referring veterinarian consisted of benazepril and furosemide and was complemented by clopidogrel. The pet owners declined any further clinical diagnostics and therapy. The dog died 19 days later. This is the first reported case of a dog with a free-floating left atrial thrombus. Specific therapeutic strategies for intracardiac thrombi in the dog are currently not available, and therefore, every patient should be treated individually.

## 1. Introduction

Cardiac thrombi in dog and humans are uncommon, in contrast to cats, where they are seen more frequently associated with cardiomyopathies [1]. Recognized risk factors in humans are mitral stenosis [2], atrial fibrillation, and mitral valve replacement [3] as well as rheumatic mitral valve diseases [4].

In cats, cardiomyopathies, left atrial enlargement, and left atrial resp. left ventricular dysfunction are discussed as risk factors for the development of intracardiac thrombi [1, 5]. Dogs with a variety of heart conditions that are accompanied by an enlarged left atrium, systolic dysfunction of the left ventricle, or cardiac arrhythmia (e.g., atrial fibrillation) are at higher risk for developing a thrombus and a resulting arterial thromboembolism [6]. In addition to that, another study reports the increased tendency for hypercoagulability during congestion within mitral valve insufficiency [7].

More than 160 years ago, Rudolf Virchow already postulated the Virchow triad describing three central factors in the pathogenesis of thrombus formation [8]:

1. Alterations of the vascular wall
2. Changes of the blood flow
3. Altered blood composition

Nowadays, this original triad is completed by a damage or a dysfunction of the endothelium or endocardium, abnormal blood stasis, abnormal haemostasis, platelets, and fibrinolysis [9]. To date, it is acknowledged that atrial fibrillation complies with the Virchow triad and therefore presents a prothrombotic state.

Only very few case reports are available on cardiac thrombi in dogs. A case study reporting atrial fibrillation in three dogs of different breeds found a thrombus in the left auricula in two of the dogs using echocardiography. The third dog was diagnosed with a thrombus located in the terminal branching of the abdominal aorta which led to occlusion of the left external iliac artery [6]. In a study by Murray et al. [10], three dogs got a pacemaker implantation because of an idiopathic third-degree atrioventricular block. A single stimulation electrode was placed in the right ventricle. All dogs developed a cranial vena cava thrombosis 26-45 months post implantation. Two of these dogs showed thrombi that reached within the right atrium. Hildebrandt et al. reported the development of thrombi at the stimulation electrode itself as an additional complication [11]. Here, two dogs were treated with a dual-chamber pacemaker and both developed a small thrombus at the electrode in the right atrium.

To the authors' knowledge, there are only two case studies available for left atrial thrombi in the dog [12, 13]. The case of an oscillating atrial thrombus in dogs as presented here is not described at all.

## 2. Case Details

A 14-year-old intact female mixed-breed dog (body weight of 8.5 kg) was presented with dyspnoea. This condition had persisted for eight days and was treated by the referring veterinarian with antibiotics, prednisolone, and vitamins. On the following day, prednisolone was substituted by a single dose of dexamethasone. Six days before presentation in our clinic, benazepril (0.3 mg/kg, PO, SID) and furosemide (1.2 mg/kg, PO, BID) were prescribed additionally. The bitch was regularly vaccinated and dewormed, with the last deworming dating three months back. The dog had never been abroad. For three years, a generalized alopecia was apparent without any known cause because the owners had declined a thorough dermatological examination; only the head and the distal limbs showed hair growth. The last period of heat was observed three years ago by the owners. For around eleven months, the dog showed polyuria und polydipsia. Food intake and defecation were reduced during the last days prior to presentation. On physical examination, the dog showed a calm and attentive general condition in upright posture. Rectal temperature was 36.8°C; mucous membranes were pink and moist with a capillary refill time of one second. Palpation of the peripheral lymph nodes revealed slightly enlarged, relocatable, and unpainful mandibular lymph nodes. In addition, a senile cataract on both eyes was diagnosed. Heart rate was 120 beats/min, and during auscultation, the volume of the heart sounds was unequal in every heartbeat. The femoral pulse was palpable on both sides, and no pulse deficit was found. The respiratory rate was 24 breaths/min and abdominally intensified. During lung auscultation, slightly amplified breathing sounds were noticed. The abdomen was prominent and indolent. Based on anamnesis and clinical examination, the preliminary diagnosis of a hyperadrenocorticism was assumed.

**Figure 1:**
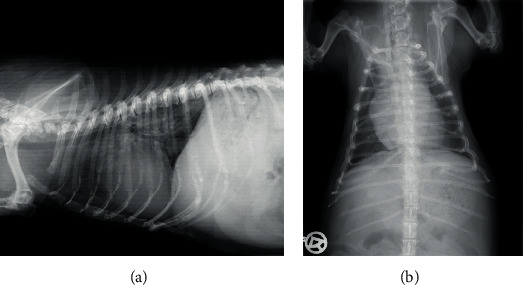
Chest X-ray in 2 planes showing cardiomegaly with left atrial enlargement (VHS 11) and mixed bronchoalveolar lung markings perihilar and in the caudodorsal lung field.

A chest X-ray in two planes was performed (Figure 1).

**Figure 2:**
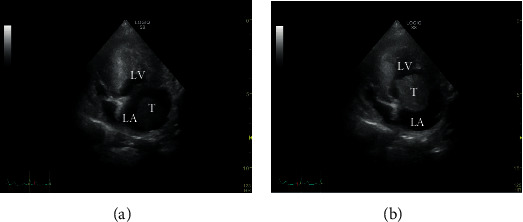
Transthoracic echocardiography (a). The thrombus in the left atrium shifts during diastole into the ostium of the mitral valve (b). T: thrombus; LA: left atrium; LV: left ventricle.

The transthoracic echocardiography (LOGIQ S8, General Electric, Munich, Germany) showed a moderate dilated left atrium (left atrium (LA): 2.66 cm, aorta (AO): 1.39 cm, LA/AO: 1.91, normal range: <1.6). A round, hyperechoic mass with a maximum diameter of 1.63 cm was floating from the left atrium into the mitral valve leaflets (Figure 2). At that time, an obstruction of the mitral valve was apparent. A notable connection between this mass and the atrial wall could not be depicted. Hence, a left atrial thrombus was suspected. A neoplasia was considered as a differential diagnosis. In addition to the mass in the left atrium, also a moderate mitral regurgitation, a low-grade pulmonary insufficiency, and a minimal aortic and tricuspid insufficiency were observed. Systolic blood pressure was measured by a Doppler device (Eickemeyer® Doppler, Tuttlingen, Germany) and was 80 mmHg.

An ACTH stimulation test was performed. The basal serum cortisol level was 9.1 *μ*g/dL (normal range: <0.9-4.5 *μ*g/dL), and the post-ACTH serum cortisol level was 61.6 *μ*g/dL. Cortisol levels after stimulation of >20 *μ*g/dL indicate a hyperadrenocorticism.

Additional diagnostics like hematology and chemistry panels, an abdominal ultrasonography, and follow-up blood pressure and echocardiographic control were refused by the owners. The prescribed therapy with benazepril (0.3 mg/kg, PO, SID) and furosemide (1.2 mg/kg, PO, BID) was supplemented with clopidogrel (2 mg/kg, PO, SID).

After 19 days, the owner reported the sudden death of their dog.

## 3. Discussion

To the best of the authors' knowledge, this is the first time that a free-floating thrombus in the left atrium of a dog is described.

The transthoracic echocardiography showed an undulating mass in the left atrium. The differential diagnosis consists of thrombus or neoplasia. Nevertheless, cardiac neoplasms are rarely seen in dogs and cats [14, 15]. The latter authors report an incidence of 0.12% to 4.33%. Hemangiosarcoma are the most commonly occurring primary neoplasms in the dog and are usually seen in the right atrium [14]. Other neoplasms to be considered are chemodectoma, chondrosarcoma, lymphoma, or ectopic thyroid carcinoma [15]. Furthermore, secondary neoplasia and metastases of extracardial tumors should be taken into account. Several case studies report left atrial neoplasms which may cause mitral valve obstruction [16–18]. In the mentioned studies, the tumor mass evolved from the left atrial wall. Here, the echocardiographical mass moved freely in the lumen of the left atrium and showed no detectable connection to surrounding structures. Therefore, the diagnosis of a left atrial thrombus was very likelier than a neoplastic occurrence.

The thrombus diagnosis was further supported by the present advanced mitral valve disease with left atrial dilation. In the context of a mitral insufficiency, regurgitation into the left atrium can appear and cause endocardial damage, the so-called jet lesions [19]. According to the Virchow's triad, this may be the starting point for a thrombus formation. Enlargement of the left atrium and congestion within mitral valve insufficiency enhance the tendency for hypercoagulability [6, 7].

This is complemented by the fact that other noncardiac diseases which tend to increase coagulability are possible causes for thrombus formation. Protein-losing nephropathy (PLN), systemic hypertension, hypothyroidism, neoplasia, and hyperadrenocorticism were named comorbidities in the context of aortic thrombosis [20]. Addressing this, the American College of Veterinary Emergency & Critical Care (ACVECC) has published a consensus in 2019. In case of an immune-mediated hemolytic anemia, PLN, or renal amyloidosis, there is a high risk for the development of thrombosis. On the other hand, a low to moderate risk for thrombosis is given in the context of sepsis, severe pancreatitis, and neoplasms (here especially adenocarcinoma). In addition to that, administration of glucocorticoids possibly enhances hypercoagulable conditions [21]. Three studies using thrombelastography substantiated this by demonstrating that dogs with hyperadrenocorticism show indeed an increased coagulability [22–24]. Park et al. [23] confirmed that this increased coagulability even persists under trilostane treatment.

In our case, the anamnestic findings and the clinical examination together with the results of the ACTH stimulation test strongly support an existing hyperadrenocorticism. The dog was dosed with single doses of prednisolone 17 days, respectively, dexamethasone 16 days prior to the ACTH stimulation test. Theoretically, this medication might have influenced the test resulting in a false-positive result. A study in healthy beagles showed that the suppressive effect of a single administration of 1 mg/kg dexamethasone was not comprehensible seven days later [25]. In our case, a false-positive result of the ACTH stimulation test is very unlikely due to the long interval between prednisolone/dexamethasone administration and the ACTH stimulation test. Further internistic intervention would have been highly desirable but was rejected by the owners.

In the case presented here, the decreased systolic blood pressure is likely to be the result of an obstruction of the inflow into the left ventricle due to the oscillating mass in the left atrium and the following decreased diastolic filling. Although arterial hypotension might be a reason to discontinue benazepril, it was further given due to its antithrombotic effects [26]. Simultaneously, phonocardiography and echocardiography were used in human patients suffering from mitral stenosis combined with a free-floating left atrial thrombus. The authors of this study could demonstrate an altering intensity of the heart murmurs depending on the position of the thrombus [27]. In our patient, there was no heart murmur on auscultation but an in intensity changing heart tone providing an indication of a restriction of cardiac function and a left atrial thrombus.

Different strategies are used for the therapy and prevention of cardiac thrombi. Surgical resection is described in the literature [2, 28, 29]. By using the inflow-occlusion technique, this can also be carried out in working hearts [30]. As an alternative, thrombolytics such as alteplase, which is a genetically produced modification of the tissue-type plasminogen activator (t-PA), can be used for thrombolysis. Only a few case reports describe the usage of t-PA for thrombolysis in dogs and cats [31, 32]. It is linked with the risk of haemorrhage and high expenses. Anticoagulants and antiplatelet drugs are used more frequently. The consensus statement of the ACVECC on the rational use of antithrombotics recommends against the use of warfarin because a severe complication of the use of warfarin is internal bleeding [33]. In contrast, low molecular weight heparins (LMWH) and direct anticoagulants are considered effective and safe drugs [34]. Acetylsalicylic acid (ASS) and clopidogrel are typical antiplatelet drugs. Clopidogrel should be used rather than aspirin in dogs and cats with increased risk for thrombosis [35]. Typically used anticoagulants are unfractionated heparin (UHF), LMWH like dalteparin or enoxaparin, and recent direct factor Xa inhibitors like rivaroxaban. LMWH are given preference to UFH, and direct factor Xa inhibitors are given preference to LMWH and UFH [35]. The consensus statement of the ACVECC recommends the administration of anticoagulants in venous thrombosis and antiplatelet drugs in arterial thrombosis. In addition, the combination of anticoagulants and antiplatelet drugs is depicted but no distinct recommendation of use is stated because of low clinical evidence [35]. Hence, a clear recommendation for a specific protocol or anticoagulant in intracardiac thrombi cannot be derived from that. In this case, clopidogrel was used to inhibit further enhancement of the left atrial thrombus. Combination with LMWH or a direct factor Xa inhibitor would have been desirable, but hospital admittance of the dog was declined by the owners. In veterinary literature, the successful treatment of a six-year-old Belgian Miniature Griffon with clopidogrel (2.5 mg/kg, PO, SID) and rivaroxaban (0.68 mg/kg, PO, SID) is described. Three months after pacemaker implantation, because of third-degree AV block, the dog showed a large thrombus in the right atrium around the pacemaker electrode. In this dog, PLN was also diagnosed and the thrombus was resolved using the aforementioned medication. ASS (5 mg/kg, PO, SID) was used in the two dogs of the study of Hildebrandt et al. [11]. Likewise, a left atrial thrombus in men was cured by administering rivaroxaban [36]. Thrombi in the left ventricle and left auricle in an American Cocker Spaniel were shown by echocardiography. 60 days after treatment of the primary cardiac disease in combination with ASS (5 mg/kg, PO, SID), the thrombi were not found anymore [12]. In principle, uncoupling of intracardiac thrombi can occur at any time resulting in arterial thromboembolism which can be fatal. In our case report, the dog died peracute. Based on the discussion of our findings, atrial thromboembolism is most likely [37].

Here, the low blood pressure and the congestion would have required the use of pimobendan as well as repeated monitoring of the blood pressure. In parallel, the treatment of the hyperadrenocorticism would have been necessary. Unfortunately, the owners rejected further clinical examinations including additional diagnostics, echocardiographic controls, and a pathological examination after the dog died.

The case study presented here describes a free-floating oscillating thrombus in the left atrium of a dog. The most likely cause of this intracardiac thrombus was a combination of a hyperadrenocorticism and a preexisting heart disease resulting in an enhanced coagulability.

Several lessons are to be learned from this case. First of all, specific testing like ACTH stimulation test or low-dose dexamethasone suppression test should be carried out once anamnesis and clinical examination point to hyperadrenocorticism. Any underlying disease that increases the risk for thrombosis should be treated immediately in dogs with cardiac diseases. For each patient, an individual risk assessment is necessary and the prophylactic medication using oral anticoagulants or inhibitors of platelet aggregation should be taken into consideration.

## Data Availability

Data are available on request from the corresponding author.

## References

[1] Fuentes V. L. (2012). Arterial thromboembolism. *Journal of Feline Medicine and Surgery*.

[2] Vitale M., Agnino A., Serena D. (1997). Asymptomatic large left-atrial ball thrombus. Secondary to mitral stenosis. *Texas Heart Institute Journal*.

[3] Martinelli F., Camurri N., Pederzolli N., Agostini F., Rambaldini M. (2017). Left atrial ball thrombus after edge-to-edge mitral valve repair. *Journal of Cardiology Cases*.

[4] Taha M. E., Eljack A., Ibrahim H., Roongsritong C. (2019). An unusual case of left atrial mural thrombus following aortic valve replacement. *Case Reports in Cardiology*.

[5] Schober K. E., Maerz I. (2006). Assessment of left atrial appendage flow velocity and its relation to spontaneous echocardiographic contrast in 89 cats with myocardial disease. *Journal of Veterinary Internal Medicine*.

[6] Usechak P. J., Bright J. M., Day T. K. (2012). Thrombotic complications associated with atrial fibrillation in three dogs. *Journal of Veterinary Cardiology*.

[7] Prihirunkit K., Sastravaha A., Lekcharoensuk C., Chanloinapha P. (2014). Hemostatic markers in congestive heart failure dogs with mitral valve disease. *Journal of Veterinary Medicine*.

[8] Anning S. T. (1957). The historical aspects of venous thrombosis. *Medical History*.

[9] Watson T., Shantsila E., Lip G. Y. H. (2009). Mechanisms of thrombogenesis in atrial fibrillation: Virchow’s triad revisited. *Lancet*.

[10] Murray J. D., O’Sullivan M. L., Hawkes K. C. E. (2010). Cranial vena caval thrombosis associated with endocardial pacing leads in three dogs. *Journal of the American Animal Hospital Association*.

[11] Hildebrandt N., Stertmann W. A., Wehner M., Schneider I., Neu H., Schneider M. (2009). Dual chamber pacemaker implantation in dogs with atrioventricular block. *Journal of Veterinary Internal Medicine*.

[12] Caivano D., Birettoni F., Giorgi M. E., Porciello F. (2014). What is your diagnosis? Intracardiac thrombus. *Journal of the American Veterinary Medical Association*.

[13] Tashjian R. J., McCoy J. R. (1960). Acquired mitral stenosis resulting in left atrial dilatation with thrombosis. A case report. *The Cornell Veterinarian*.

[14] Aupperle H., März I., Ellenberger C., Buschatz S., Reischauer A., Schoon H.-A. (2007). Primary and secondary heart tumours in dogs and cats. *Journal of Comparative Pathology*.

[15] Treggiari E., Pedro B., Dukes-McEwan J., Gelzer A. R., Blackwood L. (2017). A descriptive review of cardiac tumours in dogs and cats. *Veterinary and Comparative Oncology*.

[16] Asakawa M. G., Ames M. K., Kim Y. (2013). Primary cardiac spindle cell tumor in a dog. *The Canadian Veterinary Journal*.

[17] Buchanan J. W., Boggs L. S., Dewan S., Regan J., Myers N. C. (1998). Left atrial paraganglioma in a dog: echocardiography, surgery, and scintigraphy. *Journal of Veterinary Internal Medicine*.

[18] Fernandez-del Palacio M. J., Sanchez J., Talavera J., Martínez C. (2011). Left ventricular inflow tract obstruction secondary to a myxoma in a dog. *Journal of the American Animal Hospital Association*.

[19] Fox P. R. (2012). Pathology of myxomatous mitral valve disease in the dog. *Journal of Veterinary Cardiology*.

[20] Winter R. L., Budke C. M. (2017). Multicenter evaluation of signalment and comorbid conditions associated with aortic thrombotic disease in dogs. *Journal of the American Veterinary Medical Association*.

[21] deLaforcade A., Bacek L., Blais M.-C., Goggs R., Lynch A., Rozanski E. (2019). Consensus on the rational use of antithrombotics in veterinary critical care (CURATIVE): domain 1-defining populations at risk. *Journal of Veterinary Emergency and Critical Care (San Antonio, Tex.)*.

[22] Pace S. L., Creevy K. E., Krimer P. M., Brainard B. M. (2013). Assessment of coagulation and potential biochemical markers for hypercoagulability in canine hyperadrenocorticism. *Journal of Veterinary Internal Medicine*.

[23] Park F. M., Blois S. L., Abrams-Ogg A. C. G. (2013). Hypercoagulability and ACTH-dependent hyperadrenocorticism in dogs. *Journal of Veterinary Internal Medicine*.

[24] Rose L., Dunn M. E., Bédard C. (2013). Effect of canine hyperadrenocorticism on coagulation parameters. *Journal of Veterinary Internal Medicine*.

[25] Kemppainen R. J., Sartin J. L., Peterson M. E. (1989). Effects of single intravenously administered doses of dexamethasone on response to the adrenocorticotropic hormone stimulation test in dogs. *American Journal of Veterinary Research*.

[26] Dézsi C. A., Szentes V. (2016). Effects of angiotensin-converting enzyme inhibitors and angiotensin receptor blockers on prothrombotic processes and myocardial infarction risk. *American Journal of Cardiovascular Drugs*.

[27] Warda M., Garcia J., Pechacek L. W., Massumkhani A., Hall R. J. (1985). Auscultatory and echocardiographic features of mobile left atrial thrombus. *Journal of the American College of Cardiology*.

[28] Miura K., Ikegami Y., Momiyama Y. (2017). Syncope due to a free-floating left atrial thrombus. *Internal Medicine*.

[29] Worley D. R., Orton E. C., Kroner K. T. (2016). Inflow venous occlusion for intracardiac resection of an occluding right ventricular tumor. *Journal of the American Animal Hospital Association*.

[30] Verbeke F., Binst D., Stegen L., Waelbers T., Rooster H., van Goethem B. (2012). Total venous inflow occlusion and pericardial auto-graft reconstruction for right atrial hemangiosarcoma resection in a dog. *The Canadian Veterinary Journal*.

[31] Bliss S. P., Bliss S. K., Harvey H. J. (2002). Use of recombinant tissue-plasminogen activator in a dog with chylothorax secondary to catheter-associated thrombosis of the cranial vena cava. *Journal of the American Animal Hospital Association*.

[32] Welch K. M., Rozanski E. A., Freeman L. M., Rush J. E. (2010). Prospective evaluation of tissue plasminogen activator in 11 cats with arterial thromboembolism. *Journal of Feline Medicine and Surgery*.

[33] Blais M.-C., Bianco D., Goggs R. (2019). Consensus on the rational use of antithrombotics in veterinary critical care (CURATIVE): domain 3-defining antithrombotic protocols. *Journal of Veterinary Emergency and Critical Care (San Antonio, Tex.)*.

[34] Sharp C. R., deLaforcade A. M., Koenigshof A. M., Lynch A. M., Thomason J. M. (2019). Consensus on the rational use of antithrombotics in veterinary critical care (CURATIVE): domain 4-refining and monitoring antithrombotic therapies. *Journal of Veterinary Emergency and Critical Care (San Antonio, Tex.)*.

[35] Goggs R., Bacek L., Bianco D., Koenigshof A., Li R. H. L. (2019). Consensus on the rational use of antithrombotics in veterinary critical care (CURATIVE): domain 2-defining rational therapeutic usage. *Journal of Veterinary Emergency and Critical Care*.

[36] Gaznabi S., Abugroun A., Mahbub H., Campos E. (2019). Successful resolution of a large left atrial and left atrial appendage thrombus with rivaroxaban. *Case Reports in Cardiology*.

[37] Lozada Miranda B., Walton R., LeVine D. N., Blong A., Ware W., Ward J. (2019). Use of rivaroxaban for treatment of cranial vena cava syndrome secondary to transvenous pacemaker lead thrombosis in a dog. *Journal of Veterinary Cardiology*.

